# Clavicle Fracture Site Surgical Contouring: A Case Report

**DOI:** 10.1055/s-0043-1775880

**Published:** 2024-02-07

**Authors:** Annachiara Cavaliere, Vincenzo Cepparulo, Giuseppe Pezone, Fabrizio Schonauer

**Affiliations:** 1 Unit of Plastic Surgery, University “Federico II”, Naples, Italy

**Keywords:** case report, clavicle fracture, clavicle contour, step deformity

## Abstract

Clavicle fractures are frequent injuries accounting for approximately 4% of all fractures in adults with about 35% occurring in the shoulder region among which midshaft fractures are the most common (>66%). Nonsurgical management is the treatment of choice for most clavicle fractures; however, poor functional and aesthetic outcomes may result from nonunion, symptomatic malunion, and aesthetic impairment which are the most common complications. A young woman was referred to our clinic for a “Step Deformity” resulting after primary, nonsurgical treatment of a midshaft clavicle fracture. Residual deformity was corrected with a novel simple and little invasive approach. Midshaft clavicle fractures typically only require conservative nonsurgical treatment, nevertheless suboptimal outcomes may occur. Selective osteotomies and fixation are deemed too invasive when only cosmetic impairment of the clavicle contour is present without any functional or sensitive damage and most patients are discouraged from undergoing surgery. Thus far, no specific focus on this topic, nor exploration of possible correction can be found in the published literature. These residual deformities may be very noticeable sometimes and cause psychological distress and social life impairment. Despite no related functional impairment, this deformity should still be addressed, to improve patients' quality of life.

## Introduction

Clavicle fractures account for approximately 4% of all fractures in adults and circa 35% of all fractures are in the shoulder region. Their incidence encompasses a bimodal age distribution with young patients under 30 years of age and elderly patients over 70 being the most commonly affected. 1

Clavicle fractures are most often the result of a direct trauma impacting the shoulder laterally thereby provoking a compressive force acting along the clavicle shaft. 2

The most commonly referred classification for clavicle fractures is that proposed by Allman 3 : type 1: midshaft or middle third fractures, type 2: lateral or distal third fractures, and type 3: medial or proximal third fractures. Midshaft fractures are the most common among clavicle fractures representing nearly 80%. 4 This prevalence can be explained by the fact that the midshaft is the thinnest part of the clavicle and is not reinforced by any ligament or muscular insertion and is therefore most vulnerable to fractures. 5

Diagnosis of a clavicular fracture relies on a focused physical examination and radiographic evaluation. In most cases, nonsurgical management is the treatment of choice for midshaft clavicle fractures, resulting in complete bone union in 95% of reported cases. 6 The most common nonsurgical treatment consists of a sling positioned around the shoulders in a figure of 8, for 8 to 12 weeks. 7

However, good functional and aesthetic outcomes are not always achieved as a certain degree of deformity can frequently persist.

Surgical treatment is usually reserved for cases of associated neurovascular injuries, open fractures, multifragmented fractures, or in cases of “floating shoulder” deformity. A variety of surgical approaches have been described in the literature; however, today the “opening reduction and internal fixation” is the treatment of choice, especially in the pediatric population. 6

Notwithstanding its high frequency, several complications associated with both the surgical and nonsurgical approach to clavicle fractures have been reported. 8

One of the most common complications of the nonsurgical approach is aesthetic impairment which is usually the result of a hypertrophic bone callus formation.

Other complications are malunion and nonunion of the fracture, limited range of motion and neurovascular injuries. 8

Despite the high incidence of cosmetic clavicle contour defects resulting from a conservative treatment of midshaft clavicle fractures, thus far no specific focus on this topic, nor exploration of possible correction can be found in the published literature.

Herein we present the case of a young woman referred to our private clinic for a “step-deformity” resulting after primary, nonsurgical treatment of a midshaft clavicle fracture resulting in a residual deformity which we proceeded to treat with a little invasive approach. This case report has been reported according to the SCARE criteria. 9

## Case

**Fig. 1:**
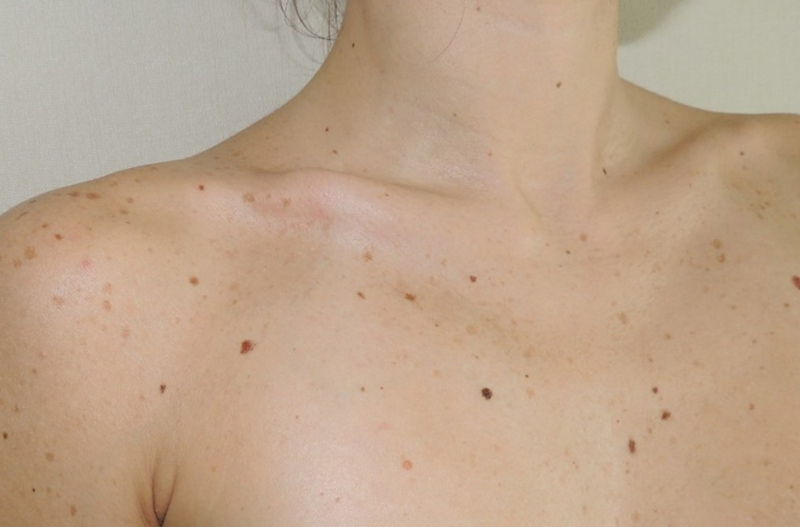
Noticeable “step deformity” of the right clavicle.

A 29-year-old patient was referred to our Plastic Surgery Clinic for a right clavicle “step-deformity” resulting after a midshaft clavicle fracture occurring 12 months earlier ( Fig. 1 ).

After an accurate clinical examination, the patient showed no functional symptomatic impairment, but reported severe psychological distress, due to the presence of her clavicle deformity. The patient complained of the appearance of her irregular and prominent right clavicle contour, particularly visible when wearing a bathing suit or low-cut clothing.

**Fig. 2:**
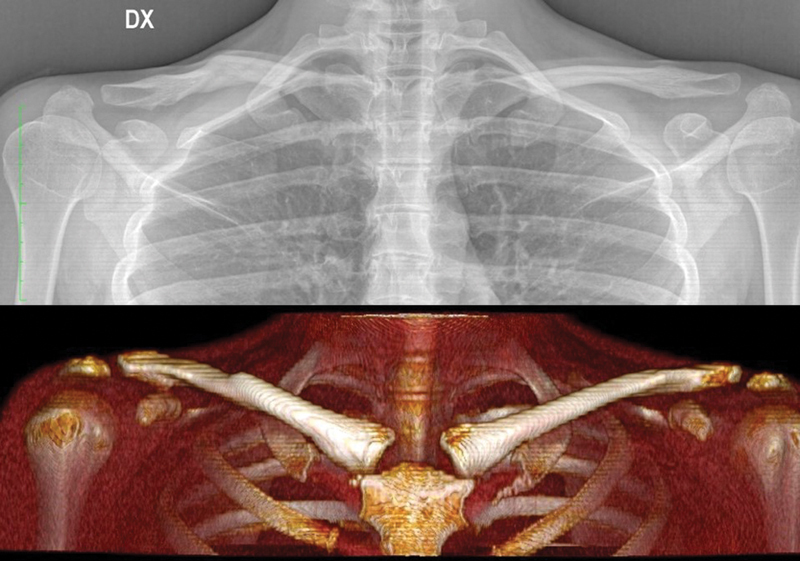
Preoperative X-ray and CT scan 3D reconstruction. 3D, three-dimensional; CT, computed tomography.

A chest X-ray and a Thorax computed tomography (CT) scan with 3D reconstructions were performed to plan a tailored approach to correct her deformity and restore a normal clavicle contour ( Fig. 2 ).

All the risks and the benefits of corrective surgery were thoroughly discussed with the patient, and specific written informed consent was signed.

**Fig. 3:**
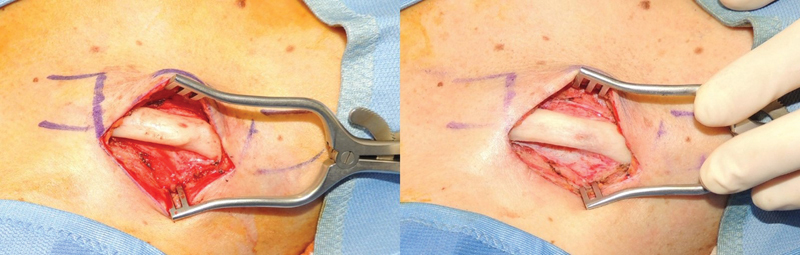
Clavicle contour before and after drilling.

Under general anesthesia, an incision was made from cranial to the upper edge of the right clavicle so that the scar would be concealed within the normal shadows of the shoulder once healed. Clavicle bone was exposed with a subperiosteal approach. Once the clavicle midshaft was completely exposed, a high-speed diamond drill (Stryker, MI) was used to smooth out the “step-deformity” and harmonize the clavicle contour. Attention was paid to avoid excessive thinning of the clavicle ( Fig. 3 ).

Once a satisfactory result was obtained, the periosteum was closed with a synthetic absorbable coated suture (4/0 Vicryl), the superficial layers with a monofilament synthetic absorbable suture (Monocryl 3/0), and running subcuticular sutures made with a monofilament suture (4/0 Monocryl) to close the skin defect. Compressive dressing was then applied.

**Fig. 4:**
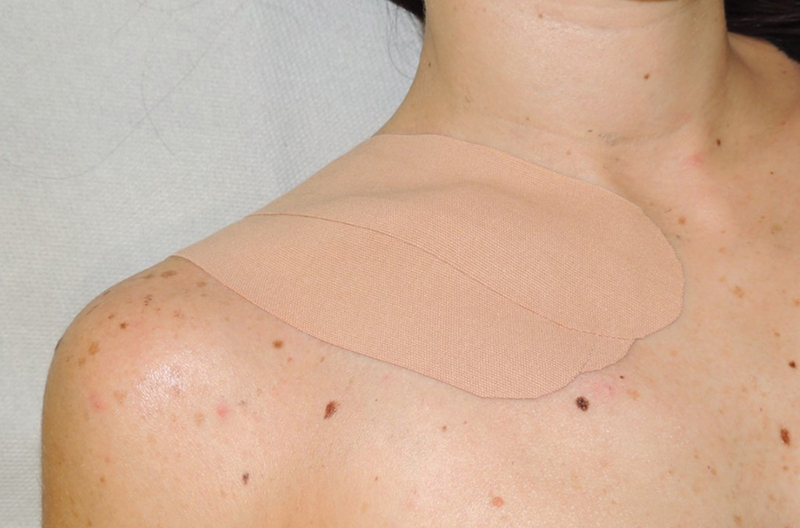
Postoperative dressing with Kinesio Taping.

**Fig. 5:**
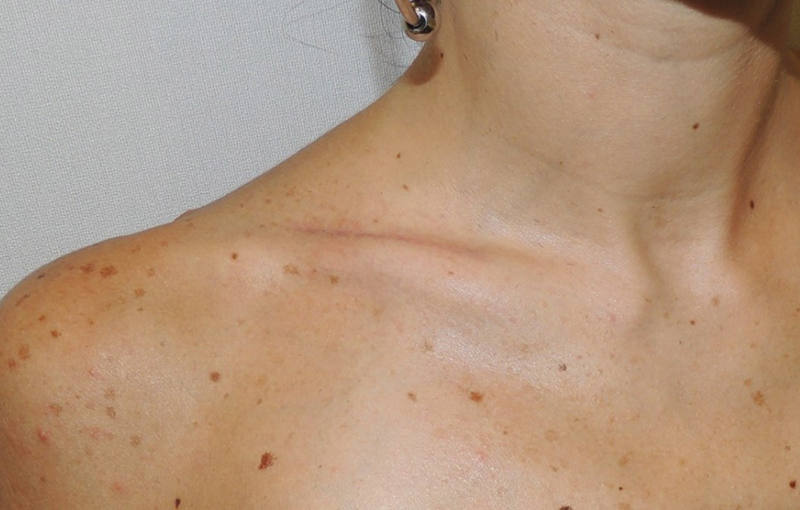
At 1-year follow-up.

The patient was given a prophylactic oral antibiotic therapy for 5 days. Taping with skin-colored kinesio tape was applied on the scar and on the new clavicle contour for 3 months after surgery. She was taught how to tape the scar at home and instructed to change it every 5 days ( Fig. 4 ). Follow-up was at 1, 3, and 6 months. At the 6-month follow-up, her clavicle contour appeared perfectly restored and symmetric to the contralateral side ( Fig. 5 ).

At 1-year postop, the patient was asked to complete the “Client Satisfaction Questionnaire-8” to evaluate her overall satisfaction with the results. 10 Questionnaire results were 32 out of 32, demonstrating a good outcome in terms of satisfaction with the appearance of her clavicle and an improvement in social and psychological impairments.

## Discussion

Clavicle fractures are frequent and account for 4 to 5% of all fractures. In most cases, the fracture involves the clavicle midshaft near the junction of the middle and distal third, while medial and distal clavicle fractures are far less common. 11

Midshaft clavicle fractures typically only require conservative nonsurgical treatment which can give satisfactory results achieving bone union with no or minimal functional impairment. Nevertheless, some authors reported a nonunion rate of up to 15 to 17% with the nonsurgical approach and even higher rates of suboptimal outcomes due to brachial plexus irritation, cosmetic deformity, and persistent local pain. As a result, approximately two-thirds of nonsurgically treated midshaft clavicle fracture patients require further surgical intervention. 12 13

Thus far, no reports in the literature have specifically focused on the aesthetic complications of treatment after the nonsurgical approach of midshaft clavicle fractures. These residual deformities, especially in young and thin women, can be very noticeable and cause discomfort with ensuing psychological distress and social life impairment.

Several different surgical approaches have been proposed to correct functional impairment after nonsurgical treatment of midshaft clavicle fractures. 14 15 16 17 18 19 20

Selective osteotomies and fixation are the most popular revision surgical techniques to address malunion, nonunion, or hypertrophic bone callus formation and to relieve compressive functional syndromes. Nevertheless, such techniques are deemed too invasive when only cosmetic impairment of the clavicle contour is present without any functional or sensitive damage and most patients are discouraged from undergoing surgery.

In patients whose only complaint is the aesthetic appearance of their clavicle, our surgical contouring technique could represent a valid option to restore normal clavicle contour after midshaft clavicular fractures. By carefully smoothing out the irregularities of the clavicle profile, almost every type of clavicle contour deformity could be addressed with a direct, little invasive approach.
